# Does Attempt at Hearing Preservation Microsurgery of Vestibular Schwannoma Affect Postoperative Tinnitus?

**DOI:** 10.1155/2015/783169

**Published:** 2015-01-12

**Authors:** Martin Chovanec, Eduard Zvěřina, Oliver Profant, Zuzana Balogová, Jan Kluh, Josef Syka, Jiří Lisý, Ilja Merunka, Jiří Skřivan, Jan Betka

**Affiliations:** ^1^Department of Otorhinolaryngology, Head and Neck Surgery, 1st Faculty of Medicine, Charles University, University Hospital Motol, V Úvalu 84, 150 06 Prague 5, Czech Republic; ^2^Institute of Experimental Medicine vvi, Institute of Auditory Neuroscience, Czech Academy of Sciences, Vídeňská 1083, 142 20 Prague 4, Czech Republic; ^3^Department of Radiodiagnostics, 2nd Faculty of Medicine, Charles University, University Hospital Motol, V Úvalu 84, 150 06 Prague 5, Czech Republic; ^4^Department of Electromagnetic Field, Faculty of Electrical Engineering, Czech Technical University, Technická 2, 166 27 Prague 6, Czech Republic

## Abstract

*Background*. The aim of this study was to analyze the effect of vestibular schwannoma microsurgery via the retrosigmoid-transmeatal approach with special reference to the postoperative tinnitus outcome. *Material and Methods*. A prospective study was performed in 89 consecutive patients with unilateral vestibular schwannoma indicated for microsurgery. Patient and tumor related parameters, pre- and postoperative hearing level, intraoperative findings, and hearing and tinnitus handicap inventory scores were analyzed. *Results*. Cochlear nerve integrity was achieved in 44% corresponding to preservation of preoperatively serviceable hearing in 47% and useful hearing in 21%. Main prognostic factors of hearing preservation were grade/size of tumor, preoperative hearing level, intraoperative neuromonitoring, tumor consistency, and adhesion to neurovascular structures. Microsurgery led to elimination of tinnitus in 66% but also new-onset of the symptom in 14% of cases. Preservation of useful hearing and neurectomy of the eighth cranial nerve were main prognostic factors of tinnitus elimination. Preservation of cochlear nerve but loss of preoperative hearing emerged as the main factor for tinnitus persistence and new onset tinnitus. Decrease of THI scores was observed postoperatively. *Conclusions*. Our results underscore the importance of proper pre- and intraoperative decision making about attempt at hearing preservation versus potential for tinnitus elimination/risk of new onset of tinnitus.

## 1. Introduction

Subjective tinnitus is a false perception of sound in the absence of an acoustic stimulation. It is the second most frequent symptom among patients with vestibular schwannoma, occurring in 63–75% of patients [1]. In about one-third of patients, it is intermittent in character. In about 10% of patients, it is the presenting symptom of vestibular schwannoma [2]. Tinnitus may be inversely proportional with tumor size. In severe form, tinnitus can lead to depressive symptoms and significantly affect quality of life [3].

The mechanisms of tinnitus origin in vestibular schwannoma are complex. Several potential mechanisms of tinnitus generation have been suggested in the literature (e.g., ephaptic coupling of cochlear nerve fibers by compression, cochlear dysfunction by ischemia or by biochemical degradation, efferent system dysfunction following compression of the nerve fibers in the inferior vestibular nerve, and cortical reorganization following hearing loss) [4, 5].

There are a limited number of studies carried out on postoperative occurrence of tinnitus in both hearing preserving and hearing nonpreserving vestibular schwannoma microsurgery [6–15]. Moreover, preoperative counseling of the patients also tends to disregard tinnitus as a possible side effect of treatment, concentrating primarily on the rates of facial nerve and hearing preservation as well as serious perioperative morbidity and mortality. Such apparent medical indifference can be partly explained by the impossibility to measure subjective symptoms and to classify them effectively. The intensity of tinnitus may be quantified subjectively by self-reporting questionnaires, such as the Tinnitus Handicap Inventory (THI) or a visual analogue scale (VAS) indexing [16, 17].

With all the advances in diagnostics and therapeutic techniques leading to reduction of morbidity and mortality, the debate on vestibular schwannoma management has centered largely on the quality of life issues. Consequently, postoperative tinnitus has increased in importance as a factor affecting the patient's quality of life [14].

This study analyzed the effect of vestibular schwannoma resection via the retrosigmoid-transmeatal approach on postoperative tinnitus. The analyses were made with special reference to the effect on tinnitus with attempted hearing preservation. In light of these results, we discuss the rationale for attempting to preserve hearing when surgically treating vestibular schwannomas.

## 2. Material and Methods

A prospective study was performed that included all 89 consecutive patients with untreated unilateral sporadic vestibular schwannoma indicated for surgical treatment via a retrosigmoid-transmeatal approach in the period from January 2008 to December 2010. All patients were operated on by the same team of two neurotologists and a neurosurgeon. Design of the study was approved by the local ethical committee.

**Figure 1:**
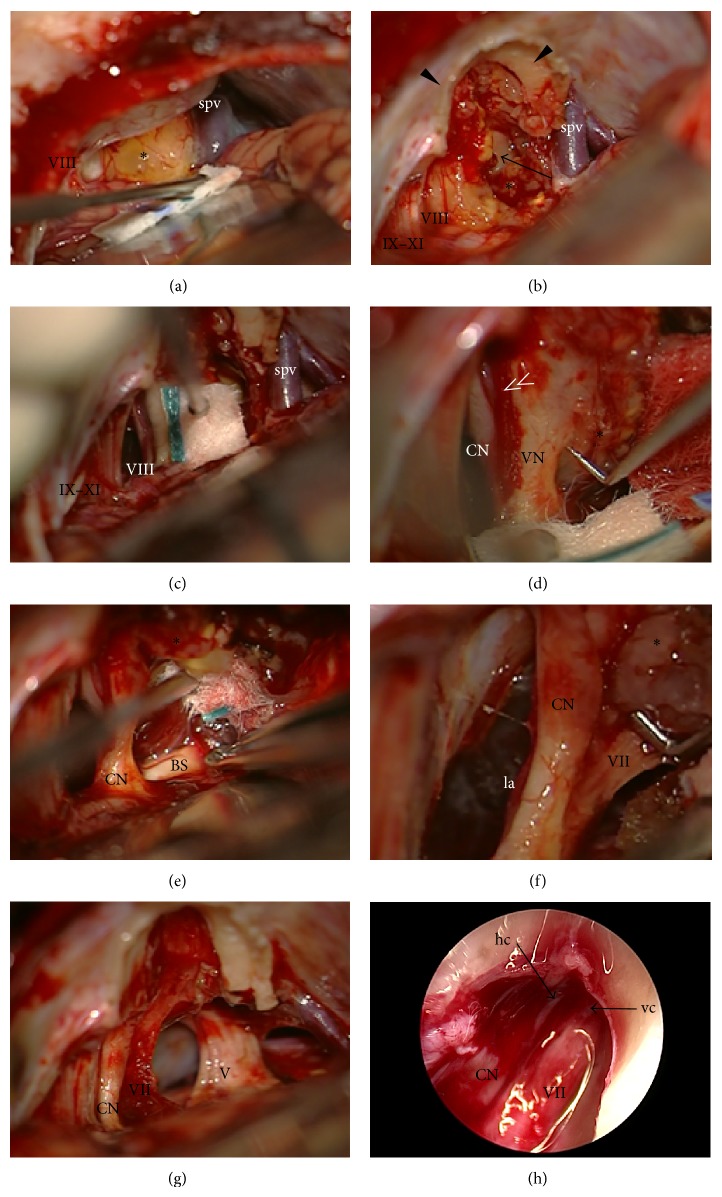
Steps of vestibular schwannoma removal via retrosigmoid-transmeatal approach (left side). (a) Tumor exposure in the cerebellopontine angle, (b) early opening of the internal auditory canal with debulking of tumor, (c) identification of CN VIII, (d) transection of vestibular nerves, (e) dissection of tumor from brainstem, (f) identification and dissection of CN VII from tumor, (g) removed tumor, and (h) endoscopic control of radicality of tumor removal in the fundus of internal auditory canal (asterisk: tumor; arrow: debulked cisternal and meatal portion of tumor; double arrowhead: arteriole on the surface of the CN VIII delineating vestibular and cochlear portion of the nerve; V: CN V, VII: CN VII, VIII: CN VIII, and IX-XI: CNs IX-XI; CN: cochlear nerve; VN: vestibular nerves; la: labyrinthine artery; spv: superior petrosal vein; hc: horizontal crest; vc: vertical crest).

Details of the surgical technique were reported elsewhere [18]; thus, only critical steps are reviewed here. All surgeries were performed in supine position with head fixation in the 3-point Mayfield clamp. Facial nerve monitoring was used to identify and confirm the function of the facial nerve in all cases and continuous brainstem auditory evoked potentials (BAEP) for hearing monitoring were employed when applicable (12 cases) (NeMo NeuroMonitor, Inomed Medizintechnik GmbH). Craniotomy has been performed exposing the edges of the transverse and sigmoid sinuses. Opened mastoid air cells were closed with bone wax. Minimally invasive approach with craniotomy ≤2.5 cm was employed for small tumors (≤20 mm extrameatal extension). Dural incision has been done along the sinuses and lateral cerebellomedullary cistern was opened to allow egress of cerebrospinal fluid (CSF). Before dural incision, controlled hypotension and assisted hyperventilation to obtain a pCO2 of about 25 mm Hg and to lower the intracranial pressure to help spontaneous cerebellar retraction have been instituted. Bolus of corticoids at the same moment could be beneficial. Mannitol infusions and lumbar drainage were not needed. Thus, a minimal brain retraction was necessary. The intrameatal tumor portion was approached by removing the posterior wall of the IAC. Any dissection of the tumor from cranial nerves and vessels was performed after adequate tumor debulking (Figure 1). In case of preoperative hearing, we attempted its preservation. On the contrary in cases of preoperative deafness we did not attempt to preserve cochlear nerve. The same would be true for cases of obvious cochlear nerve injury during tumor dissection. Endoscopic technique (rod-lens Hopkins II endoscopes 4 mm lens with 0° and 30° and 70° viewing angle and length 18 cm, Clearview, Image 1 HD three-chip camera, KARL STORZ GmbH & Co.) with standard neurotological and neurosurgical instruments was used for monitoring of neurovascular anatomy in cerebellopontine angle (CPA), during dissection of the meatal portion of tumors, to assess radicality of resection and structures of labyrinth and for identification of potential pathways for CSF-leak formation. Multiple muscle pieces and fibrin glue have been used to plug the drilled IAC after tumor removal. Dura was closed with absorbable stitches. Pieces of muscle, fascia, and fibrin glue have been used to augment duraplasty. Previously removed bone and collected bony pate have been used for reconstruction of the skull at the end of the procedure.

The data collected in each patient included the patient's age, gender, size of tumor, pre- and postoperative hearing level, intraoperative findings (e.g., cochlear nerve structural and functional preservation, radicality of tumor resection, and injury to the labyrinth), and perioperative complications. Validated questionnaires (Hearing Handicap Inventory for Adults (HHI) and Tinnitus Handicap Inventory (THI)) were employed for assessment of pre- and postoperative symptoms.

Size of the tumor was based on preoperative magnetic resonance imaging (MRI). The diameter was measured on the axial scans in the plane parallel with the long axis of the internal auditory canal (IAC) including both intra- and extrameatal portion of the tumor. Furthermore, Koos grading system was used to classify the tumor grade based on tumor extension (G1: intrameatal tumors; G2: tumors extending to the cerebellopontine angle; G3: tumors filling the cerebellopontine angle; G4: tumors compressing brainstem and cerebellum). Postoperative MRI scan to exclude tumor recurrence was undertaken at 3, 12, and 48 months postoperatively.

Facial nerve function was assessed according to the House-Brackmann grading system at discharge and on the last follow-up control (29–64 months postoperatively).

Hearing level was assessed according to the pure tone audiogram with measurement of pure tone average (PTA) and speech discrimination score (SDS). It was graded according to Gardner-Robertson's classification (GRC) (GRC1: PTA ≤ 30 dB and SDS ≥ 70%; GRC2: PTA 31–50 dB and SDS 50–69%; GRC3: PTA 51–90 dB and SDS 5–49%; GRC4: PTA > 90 dB and SDS 1–4%; GRC5: no hearing). GRC1 and GRC2 were accepted as useful hearing level.

We have analyzed the results of subjective audiometric tests (pure tone audiometry parameters: threshold measured at frequencies of 0.5, 1, 2, and 4 kHz, pure tone average (PTA); speech audiometry parameters: speech discrimination score (SDS) and speech reception threshold (SRT)). To evaluate the results of BAEP, we used the Hannover classification (H1: preserved complex of waves I, III, and V with normal or prolonged latencies of waves I–III ≤ 2.66 ms; H2: preserved complex of waves I, III, and V, but pathologically prolonged latencies of waves I–III > 2.66 ms; H3: present waves I and V, but missing wave III; H4: only one present wave, usually wave I; H5: no response).

The extent of tumor extension into the internal auditory canal was evaluated on preoperative T2-weighted MRI. The presence of fluid lateral to the tumor was considered as partial filling while absence was considered as complete filling.

The intraoperative findings that were analyzed included bleeding, tumor consistency, adhesion to neurovascular structures, presence of cystic component, and success in identification of cochlear portion of the eighth cranial nerve.

Results were analysed using chi square test, Fisher's exact test, Student's* t*-test, Tukey HSD test, and one-way ANOVA as appropriate. Analyses were performed using SPSS v16.0 (SPSS Inc., Chicago, IL, USA).

## 3. Results

All tumors were removed radically (GTR: gross total resection). We encountered 1 case of tumor recurrence on the MRI at 48 months following surgery affecting labyrinth and internal auditory canal. It was managed with stereoradiosurgery. In all the remaining cases, patients are disease-free.

There were neither infectious complications including meningitis nor perioperative mortality. The loss of continuity of the facial nerve occurred in 6% (5/89) of patients. In all cases, a direct reconstruction of the facial nerve in the cerebellopontine angle/internal auditory canal led to reinnervation (House-Brackmann grade III function). In the remaining 94% (84/89) of cases, facial nerve was preserved. Immediate postoperative excellent or very good function of the facial nerve (House-Brackmann grades I and II) was observed in 65% (58/89) of the patients. At the last follow-up, we observed excellent or very good function of the facial nerve in 67% (60/89) of cases while the severe dysfunction (House-Brackmann grade V) was present in 1% (1/89) of patients.

Eight patients had a deafened ear on the side of the tumor already preoperatively. Although the rate of cochlear nerve preservation was 44% (36/81), success rate of serviceable hearing preservation in such cases was only 47% (17/36). Overall hearing was preserved in 21% (17/81) of cases with preoperatively serviceable hearing (M/F = 11/6, 49 ± 11 years). In 79% (64/81) of patients, we demonstrated postoperative deafness (M/F = 36/28, 45 ± 14 years). Success rate of hearing preservation was dependent on the size (*P* < 0.01) and stage of the tumor (*P* < 0.01) (Table 1). Level of internal auditory canal filling was not related to the success rate of hearing preservation.

Preservation of preoperatively useful hearing was demonstrated in 21% (9/43; GRC1 = 3, GRC2 = 6) and the nonuseful hearing in 29% (8/28; GRC3 = 3, GRC4 = 5) of the patients. Moreover in two patients we observed postoperative improvement of hearing from the nonuseful to useful level. Success rate of hearing preservation was dependent on the level of preoperative hearing (*P* < 0.01) (Table 1). Accordingly, selected parameters monitored on the subjective audiometric methods correlated with the success rate of hearing preservation (*P*_0.5 kHz_ = 0.05; *P*_1 kHz_ < 0.01; *P*_2 kHz_ = 0.05; *P*_SDS_ = 0.05; *P*_SRT_ < 0.01). Also, the type of BAEP response was related to the hearing preservation (*P* = 0.03) and possibility to employ intraoperative BAEP was crucial for the outcome (*P* < 0.01). Surprisingly, preservation of BAEP response intraoperatively did not correlate with hearing preservation.

Among the key intraoperative factors affecting the chance for hearing, preservation belonged to identification of the cochlear nerve (*P* < 0.01), soft consistency of the tumor (*P* = 0.05), and the absence of adhesion of tumor to neurovascular structures (*P* < 0.01). Bleeding or presence of cystic components did not affect the success rate of hearing preservation.

HHI scores were correlated with the hearing level as assessed according to Gardner-Robertson's classification. Preoperatively, the mean HHI levels were lowest in the group of patients with useful hearing (GRC1-GRC2: 12 ± 16), followed by the group of patients with nonuseful hearing (GRC3-GRC4: 24 ± 22) and patients with nonuseful hearing/deafness (GRC5: 34 ± 17) (*P* = 0.03). Significant differences in the mean postoperative HHI level across the studied groups were not observed (GRC1-GRC2: 25 ± 18, GRC3-GRC4: 36 ± 21, and GRC5: 31 ± 22).

Overall, tinnitus occurred preoperatively in 82% (73/89) of the patients. It was slightly more prevalent in cases with preoperatively deafened ears. As such, tinnitus was reported by 88% (7/8) of patients with GRC5 while among patients with serviceable hearing it was reported in 82% (67/81). In case of preoperatively useful hearing tinnitus was observed in 74% (32/43) and in case of nonuseful hearing in 90% (34/38) of the patients (Table 2).

Tinnitus was reported by 35% (29/89) of our patients postoperatively (Table 3). Among patients reporting the symptom postoperatively, the new onset was present in 14% (4/29) (Table 4). Thus, disappearance of preoperative tinnitus was observed in 66% (48/73) of the patients (Table 5). In case of hearing preservation group, tinnitus was reported in 18% (3/17) of the cases. Furthermore, postoperative tinnitus was observed in only 11% (1/9) of patients with preserved useful hearing and in 25% (2/8) of cases with preserved nonuseful hearing. In patients with deafened ear, tinnitus was postoperatively reported in 36% (26/72) of cases.

In relation to the preoperative hearing level, postoperative tinnitus was more prevalent in patients with preoperatively serviceable hearing. It was reported by 35% (15/43) of patients with preoperatively useful hearing, by 34% (13/38) of patients with nonuseful hearing, and by only 12% (1/8) patients with preoperatively deafened ear (Table 3).

Perception of postoperative tinnitus was correlated to the status of preservation of the cochlear nerve and postoperative hearing level (*P* = 0.05) (Table 6). Highest prevalence of postoperative tinnitus was observed in cases with anatomically preserved cochlear nerve but postoperatively deafened ear. Such situation was encountered in 58% (11/19) of patients. On the contrary, the lowest prevalence of postoperative tinnitus of 11% (1/9) was identified in cases with preserved useful hearing. In case of preserved nonuseful hearing, 25% (2/8) of patients reported postoperative tinnitus. Under the setting of neurectomy of the eighth cranial nerve, postoperative tinnitus occurred in 28% (15/53) of cases.

When analyzing the new onset of tinnitus, 75% (3/4) of reported cases were observed in patients with postoperative deafness and the remaining case was observed in the group of preserved nonuseful hearing (Table 4). We did not encounter any case of the newly perceived tinnitus in patients with preoperative deafness. Its occurrence was equally distributed in both preoperatively useful and nonuseful hearing groups in 5% (2/43 and 2/38, resp.). If related to the postoperative hearing level, highest prevalence was in the group of preserved nonuseful hearing in 13% (1/8). On the contrary, patients with preserved useful hearing did not perceive any new tinnitus. Overall, in the group of patients with postoperative deafness new tinnitus was reported in 4% (3/72) only.

Results were correlated to the success rate of cochlear nerve and hearing preservation (*P* = 0.02) (Table 6). We did not encounter any case of newly reported tinnitus in the scenario of neurectomy of the eighth cranial nerve. All cases of new tinnitus occurred when attempting to preserve cochlear nerve and hearing. In 75% (3/4) of cases, structures of the cochlear nerve were preserved but the patient lost preoperative hearing. In the remaining patient, preoperatively useful hearing deteriorated to the nonuseful level.

Elimination of tinnitus was observed in 75% (6/8) of preoperatively deafened, in 61% (23/38) of nonuseful hearing, and in 44% (19/43) of useful hearing ears. If correlated with postoperative hearing level, tinnitus disappeared in 51% (37/72) of GRC5, 63% (5/8) of GRC3-GRC4, and 67% (6/9) of GRC1-GRC2.

Similarly to the previous results, disappearance of tinnitus was correlated to the status of preservation of the cochlear nerve and postoperative hearing level (*P* < 0.01) (Table 6). Highest prevalence of tinnitus elimination was observed in cases of the eighth cranial nerve section. Under such scenario, tinnitus disappeared in 86% (31/36) of cases. The worst situation was reported by patients in whom the cochlear nerve was preserved but preoperative hearing has been lost by the surgery when tinnitus was eliminated in 32% (6/19) only. In case of preservation of useful hearing 67% (6/9) and in case of nonuseful hearing 63% (5/8) of patients reported postoperative disappearance of tinnitus.

When THI scores were correlated with the hearing level as assessed according to Gardner-Robertson's classification, we did not observe any differences of THI scores across the studied groups preoperatively (GRC1-GRC2: 14 ± 19, GRC3-GRC4: 14 ± 18, and GRC5: 26 ± 26) and postoperatively (GRC1-GRC2: 17 ± 18, GRC3-GRC4: 16 ± 16, and GRC5: 12 ± 19). There was no relationship between the THI and HHI scores in all the studied groups pre- and postoperatively. Overall microsurgical treatment of vestibular schwannomas led to improved quality of life as related to impact of tinnitus as the mean THI scores decreased from preoperative to postoperative level (preoperative THI: 18 ± 20 versus postoperative THI: 14 ± 19). Nevertheless, the worst THI scores over 50 were observed only among patients who lost preoperative hearing and became deaf despite the cochlear nerve preservation as opposed to those who had eighth nerve neurectomy or hearing preserved (*P* = 0.01 and *P* = 0.03, resp.).

## 4. Discussion

Over the past several decades, the outcomes of patients with vestibular schwannomas have improved significantly. As mortality and major neurologic morbidity have been reduced to almost negligible levels, good quality of life postoperatively has become the generally accepted goal of management. Given that vestibular schwannomas represent histologically benign tumors and a significant proportion lose their growing potential, simple observation of oligosymptomatic tumors <2.5 cm is a well-accepted option [1, 3]. Preservation of neurological function, particularly facial nerve and cochlear nerve/inner ear function, is a principle goal of any therapeutic intervention [1, 3, 19–21]. Despite the fact that the microsurgery represents gold standard of vestibular schwannoma management, there are strong proponents of stereoradiosurgical treatment for tumors ≤2.5–3 cm in diameter [22]. Thus, microsurgery is indicated mainly in case of large tumors and deterioration of useful hearing during observation with attempt at its preservation and in case of disabling symptoms (mainly vertigo) [1, 3].

Factors affecting postoperative preservation of hearing have been described and summarized in many studies. Specific surgical approach, small tumor size, younger age of the patient, and the use of intraoperative neuromonitoring have all been implicated as main positive predictive prognostic factors [23–31].

In the current prospective study, we have analyzed the effect of vestibular schwannoma resection via the retrosigmoid-transmeatal approach on postoperative hearing preservation and tinnitus. The analyses were made with special reference to the effect on tinnitus of attempted hearing preservation versus nonhearing preservation surgery.

There is abundant evidence that preoperative hearing level and tumor size are the most crucial factors of success in hearing preservation. Thus, hearing preservation is advised by some experts only for cases of useful hearing and tumors extending less than 2 cm into the cerebellopontine angle [20]. Hearing preservation in large tumors ≥ 2 cm is reported to have a low success rate. However, other authors have attempted hearing preservation surgery even in patients with large tumors. These authors report a hearing preservation rate between 9 and 28% of patients [19, 25–34]. A similar strategy is adopted by our team to attempt hearing preservation when serviceable hearing is present. Such approach gives perspective for improvement of surgical results in terms of success rate of hearing preservation and is also critical for teaching purposes. Moreover in case of structurally preserved cochlear nerve despite loss of hearing there is chance for hearing rehabilitation with cochlear implantation.

In our study of 89 consecutive patients undergoing vestibular schwannoma resection via retrosigmoid-transmeatal approach, we were able to preserve cochlear nerve in 44% of cases. This result corresponds to preservation of preoperatively serviceable hearing in 47% and more specifically preoperatively useful hearing in 21%. Such data must be understood in the context of the size and grade of treated tumors. In our study, 80% of tumors were large (grades III and IV). In agreement with the published literature, main prognostic factors for hearing preservation were size of the extrameatal portion and stage of the tumor, preoperative hearing level, and possibility to employ intraoperative BAEP monitoring. We did not confirm the observation that level of internal auditory canal filling is crucial for the result. Besides that, tumor consistency and adhesion to neurovascular structures were important predictive factors for the result.

Concerning the HHI scores, observed handicap was correlated to the hearing level preoperatively. Postoperatively, we observed increase of handicap in all patients irrespective of the result. It is interesting that the subjectively perceived hearing handicap postoperatively was not different for the studied groups (useful hearing versus nonuseful hearing versus deafness).

We have unpublished results on patients from the same group who were rehabilitated postoperatively for the single sided deafness. In this group of patients who have decided on BAHA according to the soft-band testing and Bern benefit test, the HHI scores were lowest following the implantation. It is striking that scores were significantly better in the postrehabilitation period when compared to both postoperative period with single sided deafness and preoperative period when serviceable or even useful hearing was present. This could be at least partly explained by the alteration of sound localization and distortion leading to impaired speech intelligibility as a consequence of dysfunction of inner ear and cochlear nerve by tumor and its treatment [35, 36].

Tinnitus is a bothersome symptom for patients with vestibular schwannoma that appears to influence quality of life [12, 13]. Its origin remains unclear. Some works support peripheral origin while others support central pathophysiological origin [1, 2]. Similarly, there are various results of changes in tinnitus after microsurgery. Tinnitus can be eliminated, increase, change its character, or even arise as a new symptom. Previous studies identified mainly younger age, smaller tumor size, better preoperative hearing, normal and retrocochlear type of hearing loss, and preservation of the cochlear nerve as prognostic factors of tinnitus elimination [6–15].

Overall, in our study of patients undergoing vestibular schwannoma microsurgery via retrosigmoid-transmeatal approach, we observed disappearance of preoperative tinnitus in 66% but also new onset of the symptom in 14% of patients. We did not observe any relation with the tumor size, age, and gender of the patients. There was a trend for association of worse preoperative hearing with preoperative tinnitus. Such results correspond to some of the previously published works when hearing preserving surgery was attempted [9, 13].

Both preservation of useful hearing and neurectomy of the eighth cranial nerve emerged as the main prognostic factors of tinnitus elimination with microsurgery. On the contrary, preservation of cochlear nerve but loss of preoperative hearing was identified as the main factor for both tinnitus persistence and new-onset tinnitus. This is in contrast to the study of Kameda et al. [13] who in the series of 242 patients did not find correlation between either cochlear nerve resection or useful hearing preservation and postoperative development of tinnitus. Despite the fact that there is a logical trend to attempt cochlear nerve and hearing preservation microsurgery when applicable both because of the chance for improvement of future results with growing experience and because of the employment of modern technologies (e.g., cochlear implantation in case of single sided deafness), the impact of cochlear nerve preservation with simultaneous hearing deterioration must be critically considered.

As we did not encounter any case of the newly perceived tinnitus in patients with preoperative deafness when there was no attempt at the eighth cranial nerve preservation, we support such cases hearing/eighth cranial nerve nonpreserving surgery. Similar results were achieved in cases of useful hearing preservation but such result is hard to predict intraoperatively.

As all cases of new tinnitus occurred when attempting to preserve cochlear nerve and hearing but finally hearing deteriorated to deafness or nonuseful level, we expect important role of inner ear and cochlear nerve in the pathophysiological mechanisms of its origin. Such consequences were correlated with the worst THI scores as opposed to handicap improvement when tinnitus was alleviated with surgery. In contrast to our results, del Río et al. [14] in their study with both hearing preserving and hearing nonpreserving vestibular schwannoma surgery conclude that most patients do not report significant changes in their tinnitus status. According to this work, tinnitus should not be used as a criterion for selecting the surgical approach. Our results of impact of eighth nerve resection on the postoperative course of tinnitus rather support findings of results of nonhearing preserving translabyrinthine vestibular schwannoma microsurgery [10, 12, 37]. Furthermore, tinnitus was repeatedly shown to have a significant underestimated impact on the patient's postoperative course and quality of life [12–14, 38]. This may be even more evident under the scenario of simultaneous hearing loss and either tinnitus new onset or aggravation. Our data show that evaluation of both symptoms and their impact on quality of life should not be separated but rather coanalyzed.

Adequate preoperative discussion of potential impact of tinnitus on quality of life with vestibular schwannoma microsurgery indicated for patients seems to be crucial as well. In general severity of tinnitus correlates to psychological and general health factors and the risk of depression and insomnia may be higher in patients with tinnitus [39].

## 5. Conclusion

This study analyzed the effect of vestibular schwannoma microsurgery via the retrosigmoid-transmeatal approach with special reference to the effect on tinnitus with attempted hearing preservation. Our results show that despite the fact that the cochlear nerve integrity can be preserved in a significant number of patients even in large tumors hearing preservation is achieved only in selected cases. Main prognostic factors predicting hearing preservation are extent of tumor, preoperative hearing level, possibility of intraoperative neuromonitoring, tumor consistency, and adhesion to neurovascular structures. Overall tumor removal led to elimination of tinnitus in a significant proportion of patients. Preservation of useful hearing and neurectomy of the eighth cranial nerve emerged as the main prognostic factors of tinnitus elimination. On the contrary, preservation of cochlear nerve but loss of preoperative hearing was identified as the main factor for both tinnitus persistence and new-onset tinnitus. Our results underscore the importance of proper pre- and intraoperative decision making about attempt at hearing preservation versus potential for tinnitus elimination/risk of new onset of tinnitus.

## Figures and Tables

**Table 1:** Factors affecting hearing preservation among the patients with preoperatively serviceable hearing.

	Hearing preserved	Deafness	*P*
*N*	17	64	

Size (mm)	19 ± 9 (11–40)	29 ± 11 (9–59)	**<0.01**

Grading (Koos)	G1: 2; G2: 4; G3: 7; G4: 4	G1: 1; G2: 3; G3: 11; G4a: 45; G4b: 4	**0.02**

Preoperative hearing level	GRC1: 6; GRC2: 7; GRC3: 2; GRC4: 2	GRC1: 12; GRC2: 18; GRC3: 20; GRC4: 4	**<0.01**

Type of BAEP response	H2: 5; H3: 4; H4: 1; H5: 7	H1: 1; H2: 8; H3: 10; H4: 2; H5: 51	**0.03**

Intraoperative BAEP	8	4	**<0.01**

Soft tumor consistency	7	21	**0.03**

Tumor adhesion	4	45	**<0.01**

GRC: Gardner-Robertson's classification; BAEP: brainstem auditory evoked potentials; H: Hannover classification of BAEP response.

**Table 2:** Prevalence of the preoperative tinnitus according to the pre- and postoperative hearing level.

		Preoperative tinnitus	
Preoperative hearing level	*N*	*N *	%

GRC1-GRC2	43	32	74
GRC3-GRC4	38	34	90
GRC5	8	7	88

Postoperative hearing level	*N *	*N *	%

GRC1-GRC2	9	7	78
GRC3-GRC4	8	6	75
GRC5	72	60	83

GRC: Gardner-Robertson's classification.

**Table 3:** Prevalence of the postoperative tinnitus according to the pre- and postoperative hearing level.

		Postoperative tinnitus	
Preoperative hearing level	*N *	*N *	%

GRC1-GRC2	43	15	35
GRC3-GRC4	38	13	34
GRC5	8	1	12

Postoperative hearing level	*N *	*N *	%

GRC1-GRC2	9	1	11
GRC3-GRC4	8	2	25
GRC5	72	26	36

GRC: Gardner-Robertson's classification.

**Table 4:** Prevalence of the new postoperative tinnitus according to the pre- and postoperative hearing level.

		New tinnitus	
Preoperative hearing level	*N *	*N *	%

GRC1-GRC2	43	2	5
GRC3-GRC4	38	2	5
GRC5	8	0	0

Postoperative hearing level	*N *	*N *	%

GRC1-GRC2	9	0	0
GRC3-GRC4	8	1	13
GRC5	72	3	4

GRC: Gardner-Robertson's classification.

**Table 5:** Prevalence of resolution of preoperative tinnitus according to the pre- and postoperative hearing level.

		Elimination of tinnitus	
Preoperative hearing level	*N *	*N *	%

GRC1-GRC2	43	19	44
GRC3-GRC4	38	23	61
GRC5	8	6	75

Postoperative hearing level	*N *	*N *	%

GRC1-GRC2	9	6	67
GRC3-GRC4	8	5	63
GRC5	72	37	51

GRC: Gardner-Robertson's classification.

**Table 6:** Correlation of preservation of the anatomical and functional integrity of the cochlear nerve with preoperative, postoperative, and new postoperative tinnitus and resolution of tinnitus.

Cochlear nerve		Postoperative hearing level		Preoperative tinnitus		Postoperative tinnitus		New tinnitus		Elimination of tinnitus	
				*N*	%	*N*	%	*N*	%	*N*	%
Interrupted	53	GRC5	53	46	87	15	28	0	0	31	86
Preserved	36		19	14	74	11	58	3	16	6	32
		GRC3-GRC4	8	6	75	2	25	1	13	5	63
		GRC1-GRC2	9	7	78	1	11	0	0	6	67

GRC: Gardner-Robertson's classification.
